# Navigating the Dynamic Nature of Mitral Regurgitation With the Use of Multimodality Imaging in a Young Woman

**DOI:** 10.7759/cureus.74786

**Published:** 2024-11-29

**Authors:** Hunaina Shahab, Maryam H Khan, Nina Kukar, Patita Sitticharoenchai, Dua-Noor Butt

**Affiliations:** 1 Cardiology, Mount Sinai West Hospital, New York, USA; 2 Public Health, Johns Hopkins Bloomberg School of Public Health, Baltimore, USA

**Keywords:** mitral regurgitation, mitral valve prolapse, sedation, transesophageal echocardiography, transthoracic echocardiography

## Abstract

The mechanism and severity of mitral valve (MV) regurgitation (MR) play a critical role in guiding treatment decisions. Transthoracic echocardiography (TTE) is the primary diagnostic modality for evaluating MV disease. Discordant findings on TTE can be further quantified through transesophageal echocardiography (TEE). We describe the case of a young woman with worsening exertional dyspnea who was found to have restricted posterior MV leaflet and moderate to severe eccentric MR on TTE. TEE was subsequently performed to determine the exact mechanism of MR revealing the prolapse of the A2 segment of the MV. However, TEE significantly underestimated MR severity, downgrading it to visually mild to moderate MR and quantitatively moderate MR. This discrepancy highlights the potential for significant variation in MR severity assessment under general anesthesia, emphasizing the impact of hemodynamic loading conditions. In our case, intravenous sedatives may have altered the loading conditions reducing MR severity on TEE compared to TTE. Given her symptom severity, MV pathology, left ventricular dilatation, and the higher MR severity observed on TTE, she underwent surgical MV repair, in alignment with the Class I recommendation by the American College of Cardiology/American Heart Association (ACC/AHA) valvular heart disease guidelines. Postoperatively, she experienced significant improvement in symptoms and quality of life.

## Introduction

Mitral valve (MV) regurgitation (MR) is the most common type of heart valve disease [1]. Significant MR has important prognostic implications with mortality rates up to 16%, 31%, and 59% at 5, 10, and 20 years, respectively, from the time of diagnosis [2]. The exact mechanism and severity of MR influence treatment decisions [3]. MV surgery, particularly MV repair, is the Class 1 recommendation for the treatment of severe symptomatic primary MR, irrespective of left ventricular ejection fraction (LVEF) [4] for improvement in symptoms and heart failure. Mitral transcatheter edge-to-edge repair (TEER), which involves the coaptation of the MV leaflets, is a safe and effective, minimally invasive percutaneous intervention for patients with a favorable anatomy and symptoms from primary MR who face high or prohibitive risks with traditional surgery and have a life expectancy of at least one year [4]. For patients, who cannot undergo surgery or in whom surgery needs to be delayed, with symptomatic or asymptomatic severe primary MR with left ventricular dysfunction, it is reasonable to treat them with guideline-directed medical therapy (GDMT) [4]. Transcatheter MV replacement offers a less-invasive alternative to surgery and may address some of the challenges associated with TEER including complex MV anatomy, not suitable for TEER [5]. For patients with chronic, severe secondary MR and heart failure with reduced LVEF, guidelines recommend treatment with standard GDMT for heart failure [4]. This includes medications like ACE (angiotensin-converting enzyme) inhibitors, ARBs (angiotensin receptor blockers), beta-blockers, aldosterone antagonists, ARNI (angiotensin receptor neprilysin inhibitor), and, when appropriate, biventricular pacing [4].

Transthoracic echocardiography (TTE) is the primary diagnostic modality for MV disease. Discordant or indeterminate findings regarding the severity on TTE can be further quantified through transesophageal echocardiography (TEE), an important adjunct when more detailed visualization of the MV morphology and the left atrium is needed [1]. Guidelines also recommend the appropriate use of TEE when better visualization of relevant structures is needed and in the assessment of valvular structure and function to assess eligibility and planning of intervention [6]. 

The severity of MR can change dramatically under different hemodynamic loading conditions [7]. The reduction in MR severity is correlated with the depth of anesthesia, with a higher likelihood of MR downgrading occurring at deeper levels of sedation compared to lighter sedation [8]. It has been shown that nearly half of the patients with significant MR have a significant reduction in parameters of MR severity under general anesthesia during intraoperative TEE [9]. A recent study showed that general anesthesia caused a more pronounced reduction in the apparent severity of MR in patients with secondary MR compared to those with primary MR [10]. This dynamic nature of MR can result in challenges in the accurate diagnosis and management of MR. 

We describe the case of a young woman who presented with a history of worsening exertional dyspnea and was found to have moderate to severe eccentric MR on TTE. When TEE was done to elucidate the exact mechanism of MR, her MR severity reduced to mild to moderate which could potentially affect clinical management. The aim of this case report is to highlight the dynamic nature of MR and to show a hemodynamically and clinically oriented workflow to help guide management. 

## Case presentation

**Figure 1:**
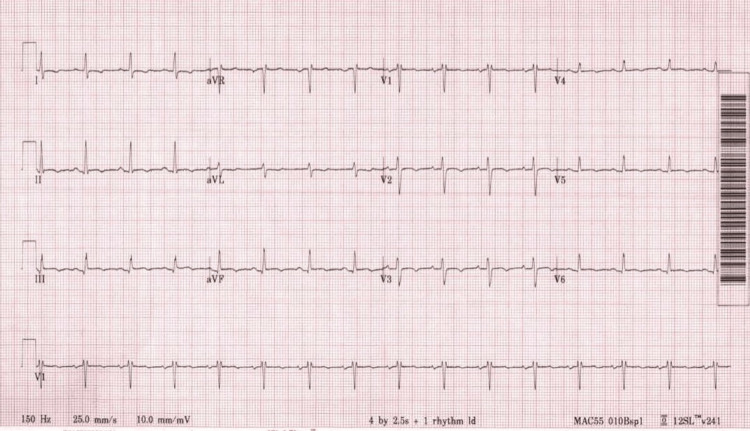
Twelve-lead electrocardiogram Normal sinus rhythm with nonspecific significant ST/T changes.

A 27-year-old woman presented to the outpatient cardiology clinic with worsening exertional dyspnea over the past six to eight months. She also experienced orthopnea, requiring the use of two pillows while sleeping, increased episodes of lightheadedness when changing positions from sitting to standing, and worsening insomnia. Her symptoms had worsened to the point of being unable to walk more than two blocks on level ground and a few steps on the stairs without dyspnea and fatigue. She denied chest pain, palpitations, or syncope. On examination, her findings included a normal jugular venous pressure, normal S1 and S2, grade 3/6 systolic murmur radiating to the axilla and the back, absence of lung crepitations, and absence of pedal edema. Her electrocardiogram (ECG) showed a normal sinus rhythm, with nonspecific ST/T changes (Figure 1).

**Figure 2:**
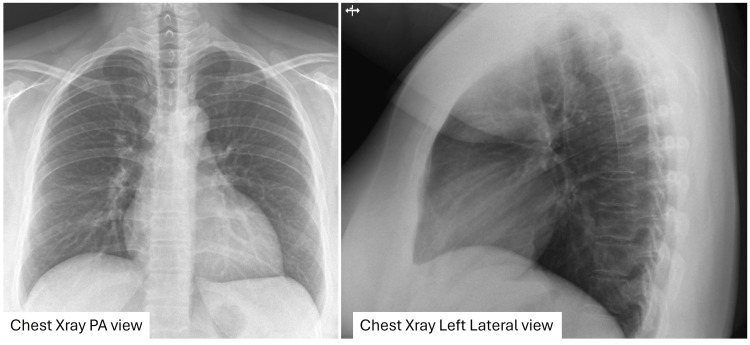
Chest X-ray PA and left lateral views showing mild cardiomegaly No evidence of pulmonary pathology was identified. PA, posteroanterior.

Her blood work, including hemoglobin, was normal. Her chest X-ray is shown in Figure 2.

Her TTE showed that the left ventricular (LV) cavity was dilated with a left ventricular end-diastolic diameter (LVEDD) of 5.3 cm with normal LV thickness, LVEF of 55-60%, restricted posterior leaflet of the MV, and visually moderate to severe eccentric, posteriorly directed, MR as shown in the parasternal long axis of TTE (Video 1). The left atrium was severely dilated with a left atrial volume index of 57 ml/m^2^. Quantitatively, the effective regurgitant orifice area (EROA) was 0.27 cm^2^, regurgitant volume was 51 ml, and regurgitant fraction was 69%. The quantitative parameters (EROA, regurgitant volume, and regurgitant fraction) indicate the severity of the MR.

**Video 1:** Transthoracic echocardiogram showing parasternal long-axis view with color Doppler Parasternal long-axis view showing a normal left ventricular ejection fraction, restricted posterior mitral valve leaflet, and posteriorly directed, eccentric, moderate to severe mitral regurgitation jet.

The focused four-chamber view (Video 2) shows the eccentric MR jet in another view.

**Video 2:** Transthoracic echocardiogram showing four-chamber focused view with color Doppler Focused four-chamber view showing another view of the posteriorly directed, eccentric, moderate to severe mitral regurgitation jet.

The three-chamber view (Video 3) highlights the significant eccentricity of the MR jet with the Coanda effect. 

**Video 3:** Transthoracic echocardiogram showing three-chamber focused view with color Doppler Focused three-chamber view highlighting the significant eccentricity of the MR jet. MR, mitral valve regurgitation.

**Figure 3:**
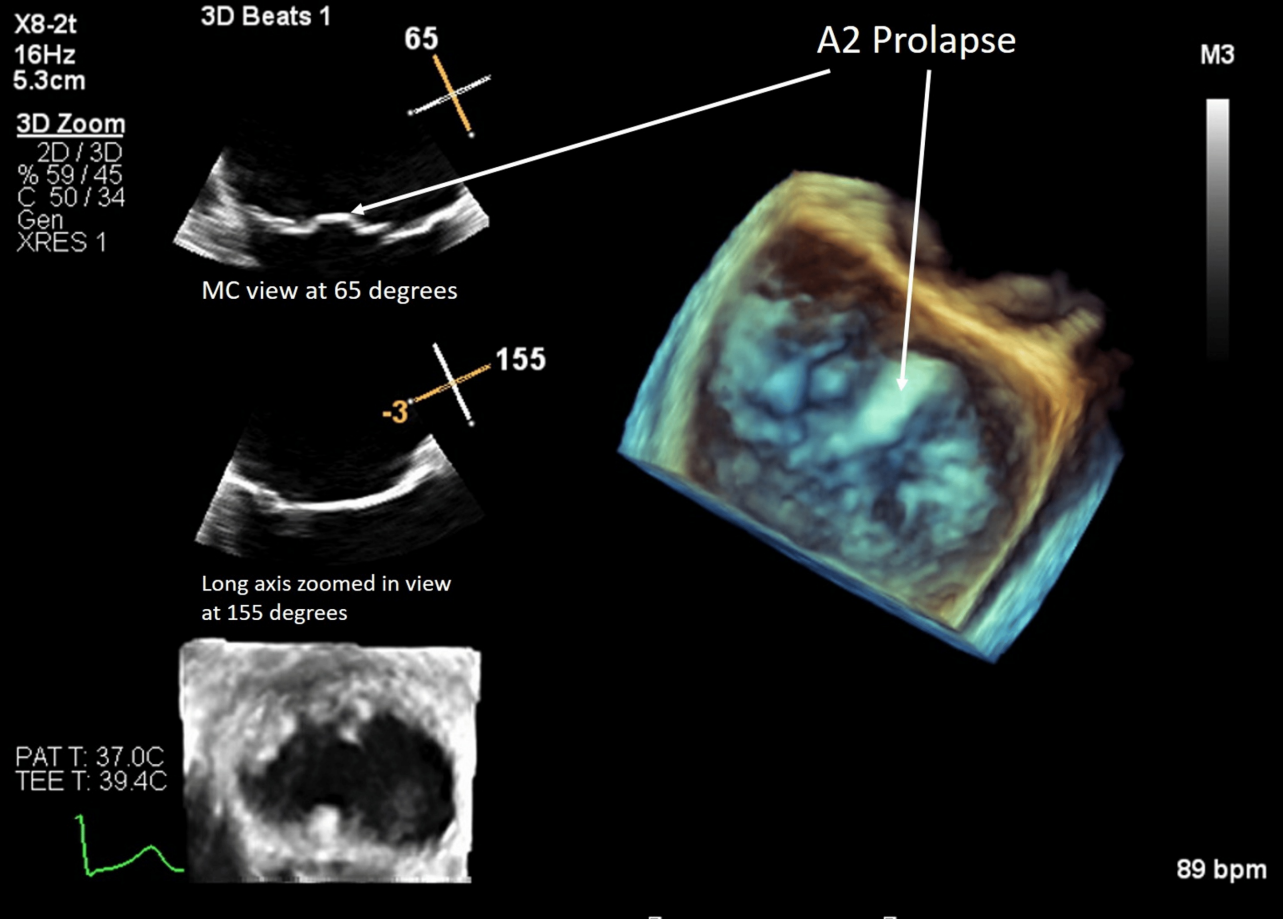
Three-dimensional TEE view of the mitral valve Three-dimensional TEE showing the mitral valve from the surgeon's view. The A2 scallop of the mitral valve is prolapsed as indicated by the white arrow. The angles of the TEE are indicated in the figure. TEE, transesophageal echocardiogram.

To accurately diagnose the mechanism of the significant MR, assess MV anatomy, and quantify MR severity, a transesophageal echocardiogram (TEE) was performed. As this was an outpatient study planned as an uncomplicated TEE, the sedation was handled by the echo lab nursing staff and the physician who performed the procedure. She received local pharyngeal anesthesia with viscous lidocaine spray applied to the oropharynx followed by intravenous (IV) midazolam and fentanyl according to the hospital protocol. The hospital follows an age-based dosage protocol for uncomplicated outpatient TEE, whereby IV midazolam is given in increments up to a maximum of 2 milligrams and IV fentanyl is given in increments up to a maximum dose of 100 micrograms, in patients between 18 and 59 years of age, titrating the dose according to patient's sedation level. In patients 60 years and above, the maximum dose of IV midazolam given is 1 milligram and IV fentanyl is 50 micrograms. Vitals were monitored before, during, and after the procedure. Her TEE showed an LVEF of 55-60%, dilated left atrium and left ventricle, restricted posterior leaflet, and also the prolapse of the MV A2 scallop as seen in Figure 3 and Video 4. 

**Video 4:** Surgeon's view of the mitral valve on three-dimensional TEE Prolapse of the A2 scallop of the anterior mitral valve leaflet. TEE, transesophageal echocardiogram.

There was visually mild to moderate eccentric, posteriorly directed MR as shown in Video 5.

**Video 5:** Four-chamber and two-chamber transesophageal views Normal left ventricular ejection fraction and mild to moderate mitral regurgitation. The angles for the TEE are noted in the video. TEE, transesophageal echocardiogram.

Additional TEE views highlighting the MR are shown in Video 6. The color Doppler baseline shift was done to optimize MR jet visualization and assist in MR quantification.

**Video 6:** Transesophageal views showing visually mild to moderate mitral regurgitation The color Doppler baseline shift was done to optimize the visualization of MR jet and to assist in quantification of MR. MR, mitral valve regurgitation.

**Figure 4:**
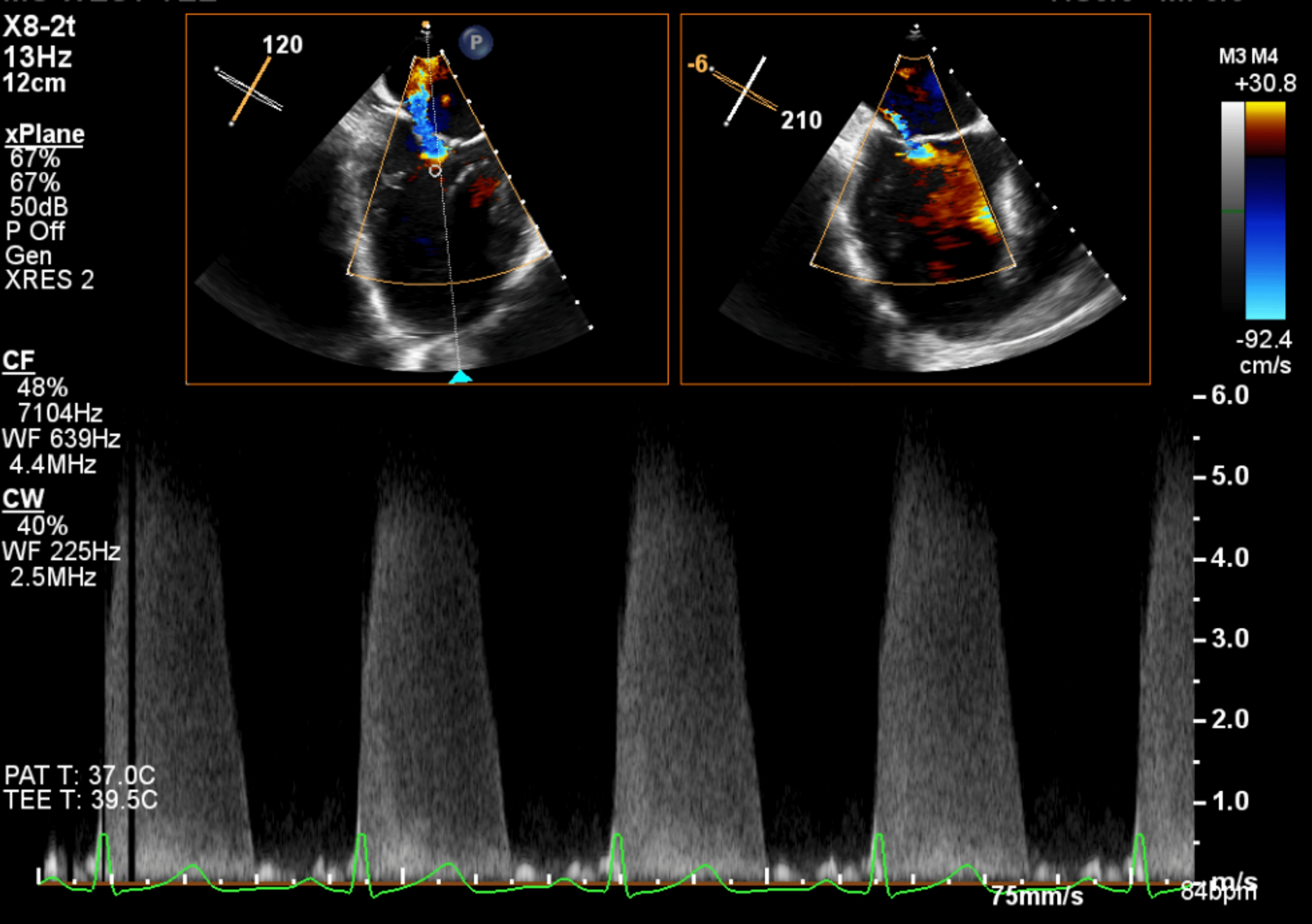
Continuous-wave Doppler evaluation of mitral regurgitation.

The continuous wave Doppler evaluation of MR at the MV is shown in Figure 4.

**Figure 5:**
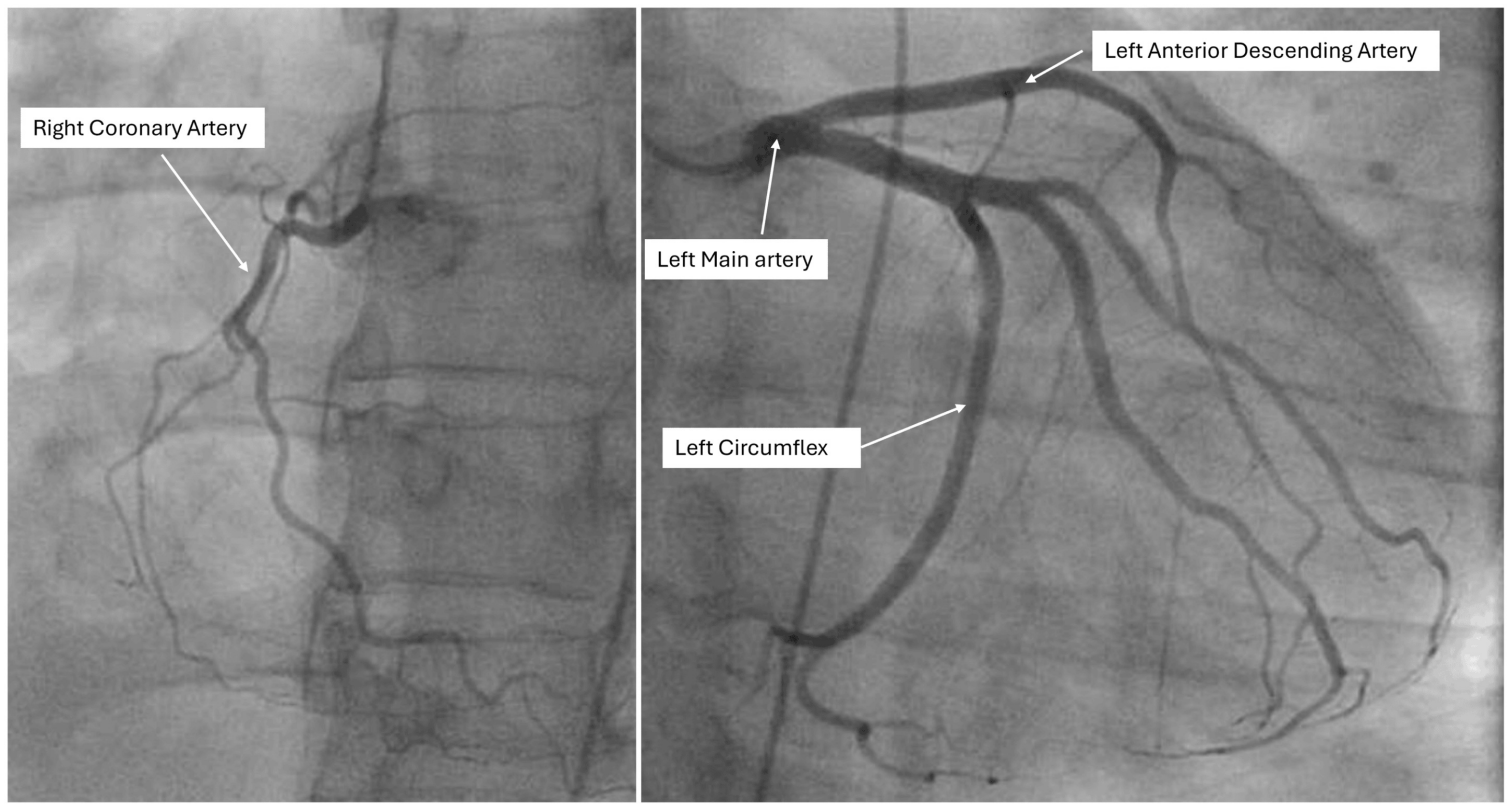
Coronary angiogram showing normal coronary arteries.

Quantitatively, the MR was moderate with EROA of 0.22 cm^2^, regurgitant volume of 38 ml, and regurgitant fraction of 51%. With the mechanism of MR noted on TEE, dilatation of the LV cavity and moderate to severe MR noted on TTE, in the presence of significant symptoms, she was referred to cardiothoracic surgery for MV intervention as per the American College of Cardiology/American Heart Association (ACC/AHA) guideline recommendations [4]. She underwent a cardiac catheterization which revealed increased right-sided pressures, mild pulmonary hypertension, (pulmonary vascular resistance index was mildly increased, pulmonary capillary wedge pressure was increased, and LV end-diastolic pressure was mildly elevated), and decreased cardiac output. A coronary angiogram showed normal coronary arteries as shown in Figure 5.

**Figure 6:**
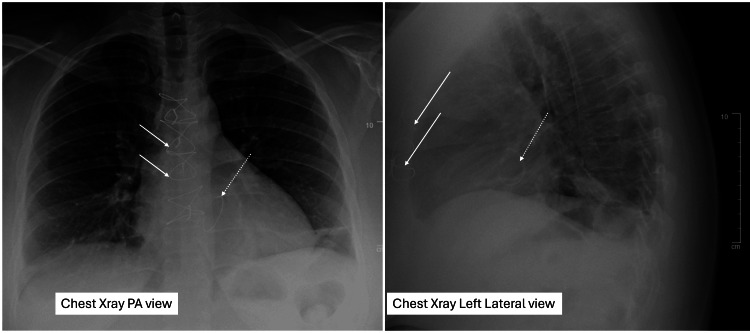
Postoperative chest X-ray showing sternal sutures (white arrows) The mitral annuloplasty ring is also in place (dotted arrows).

She subsequently underwent elective MV intervention (surgical edge-to-edge {Alfieri} MV repair) as per the ACC/AHA valvular heart disease guidelines class 1 recommendations for symptomatic MR [4]. During the procedure, a small skin incision and median sternotomy were performed and sutures were placed around the MV annulus. A true size 26 mm Physio Flex annuloplasty band was placed to encourage the coaptation and a single Magic suture was placed toward the anterior and posterior leaflets of the valve. Post-bypass TEE showed trivial MR and a normal MV gradient. This was confirmed on a repeat TTE after the procedure. The postoperative chest X-ray is shown in Figure 6. The repaired MV on the postoperative TTE is shown in Video 7. There was trivial to mild MR after the repair.

**Video 7:** Short-axis view of the mitral valve on transthoracic echocardiogram after mitral valve repair surgery. TTE, transthoracic echocardiogram.

On six months follow-up, her symptoms improved significantly and she has a good functional status.

## Discussion

TEE's proximity to the left atrium can provide an unobstructed view of the MV [11] and therefore improve the visualization of the valve's anatomy and morphology and provide an accurate quantification of the MR severity, mechanism, and important details which help in assessing reparability of the valve [6,12]. Three-dimensional (3D) imaging allows even further detailed characterization of the valve anatomy and pathology and assists in determining the eligibility criteria for repair [1]. Furthermore, there are special TEE-guided imaging techniques like the cardiac TrueVue Glass© that allow for further detailed images by peeling away layers and providing only anatomical landmarks, helping in viewing the direction of the MR jet [13]. 

In our patient, TEE helped us in further identifying the prolapse of the A2 segment of the MV; however, it significantly underestimated the severity, indicating mild-moderate MR compared to moderate-severe MR evident on the TTE. According to the ACC/AHA valvular heart disease guidelines [4], mild-to-moderate MR does not necessitate MV repair, therefore the reduction in MR severity on TEE may potentially have influenced the decision of intervention. However, recent studies have revealed significant discrepancies in the assessment of MR severity in patients who are under general anesthesia, where MR severity has been underestimated with increasing levels of sedation [14-16]. Afterload and LV contractility decrease upon the induction of anesthesia or sedation, as these agents decrease sympathetic stimulation and systemic vascular resistance through arterial and venous vasodilation [15].

The effect of general anesthesia was studied in patients with moderate-to-severe MR who had preoperative and intraoperative TEE [9]. In this study, as compared to the preoperative TEE in which patients received local pharyngeal anesthesia and IV midazolam and/or meperidine, patients during intraoperative TEE received IV midazolam, fentanyl, etomidate, and succinylcholine followed by pancuronium and inhalational isoflurane followed by intubation. It was noted that the systolic and mean arterial blood pressure and LV end-systolic and end-diastolic dimensions were reduced in intraoperative cases of TEE when compared to preoperative TEE, depicting modified loading conditions. There was a reduction in the mean color Doppler jet area and the mean vena contracta of the MR with blunting of the mean pulmonary venous flow signifying a reduction in MR severity. Approximately half of the patients in the study showed an improvement in the MR severity by one grade under general anesthesia [9]. The downgrading of MR severity was similar for primary versus secondary MR but MR due to flail leaflets was not downgraded [9,17]. Other studies have shown that the effect of general anesthesia is more pronounced on secondary MR as usually these are due to dilated mitral annulus or restricted posterior leaflet [17,18].

Our case is somewhat unique in that her preoperative TEE showed a reduction in the severity of the MR as compared to the TTE. Previously a study has shown that the severity of MR reduces with higher levels of sedation measured by a lower bispectral index, thereby the authors recommended assessing the MR severity at a shallower depth of anesthesia [8]. In another study, preprocedural TEE was taken as a baseline measurement of MR severity before measuring MR severity under general anesthesia [10]. In this study, only IV midazolam was used for sedation along with local pharyngeal anesthesia [10]. In our case, it may be possible that the administration of IV fentanyl along with midazolam may have caused deeper levels of sedation, altering the hemodynamic loading conditions which may have reduced the severity of MR during the TEE.

In such cases where the unloading effects of anesthesia may have downgraded MR and the severity is unclear, research has shown that provocative testing (preload test or afterload challenge) under TEE guidance can be used to resolve the problem and determine whether surgical intervention is indicated [17,19]. Such tests try to reproduce the same pre-anesthesia MR severity with preload and afterload challenges [17,19]. Additionally, studies have shown the benefit of using 3D TEE analysis to help analyze cases in which there is a potential downgrading of MR severity under sedation [10].

Cardiac magnetic resonance (CMR) imaging has also been shown to provide more reproducible quantitative measurements of regurgitant volume and regurgitant fraction, LVEF, and LV volumes. Recent guidelines recommend CMR to quantify MR severity when echocardiographic and clinical findings are discordant [4]. Cine CMR and other dedicated sequences allow for a detailed inspection of the MV anatomy and give an accurate quantification of MR severity and regurgitant volumes [20] and should be considered the next step in the diagnosis in cases where discrepant data hinders the correct management. In our case, however, the patient had significant symptoms that affected her quality of life, and because of the severity of MR on TTE [4], dilated LV cavity, and the known effects of sedation on MR severity [8-10], the decision to proceed for valve repair was made. Her significant symptom improvement after the surgery further strengthened the decision for valve repair.

## Conclusions

MR is a dynamic pathology, and its severity is affected by hemodynamic conditions. Our case demonstrates an improvement in MR severity during TEE which may be due to the administration of IV sedatives which may have caused deeper levels of sedation. The dynamics of MR due to volume conditions, heart rhythm, and respective medical treatment require a high level of standardization in echocardiography. With this case, we aim to show a hemodynamically and clinically oriented workflow, which integrates a detailed MR classification scheme via multimodality imaging, considering the clinical symptomatology, the chronicity of the disease process, the MV morphology, and imaging parameters characterizing the left atrial and LV remodeling to help guide management. Furthermore, CMR can be considered in cases where there is a mismatch between patient symptoms and the severity of MR noted on echocardiogram which can potentially hinder the appropriate management of MR. 

## References

[1] El Sabbagh A, Reddy YN, Nishimura RA (2018). Mitral valve regurgitation in the contemporary era: Insights into diagnosis, management, and future directions. JACC Cardiovasc Imaging.

[2] Finkelstein DM, Schoenfeld DA (1999). Combining mortality and longitudinal measures in clinical trials. Stat Med.

[3] Wang A, Grayburn P, Foster JA (2016). Practice gaps in the care of mitral valve regurgitation: Insights from the American College of Cardiology mitral regurgitation gap analysis and advisory panel. Am Heart J.

[4] Otto CM, Nishimura RA, Bonow RO (2021). 2020 ACC/AHA guideline for the management of patients with valvular heart disease: Executive summary: A report of the American College of Cardiology/American Heart Association Joint Committee on Clinical Practice Guidelines. J Am Coll Cardiol.

[5] Chiarito M, Pagnesi M, Martino EA (2018). Outcome after percutaneous edge-to-edge mitral repair for functional and degenerative mitral regurgitation: A systematic review and meta-analysis. Heart.

[6] Hahn RT, Abraham T, Adams MS (2013). Guidelines for performing a comprehensive transesophageal echocardiographic examination: Recommendations from the American Society of Echocardiography and the Society of Cardiovascular Anesthesiologists. J Am Soc Echocardiogr.

[7] Keren G, Katz S, Strom J, Sonnenblick EH, LeJemtel TH (1989). Dynamic mitral regurgitation. An important determinant of the hemodynamic response to load alterations and inotropic therapy in severe heart failure. Circulation.

[8] Chin JH, Lee EH, Choi DK, Choi IC (2012). The effect of depth of anesthesia on the severity of mitral regurgitation as measured by transesophageal echocardiography. J Cardiothorac Vasc Anesth.

[9] Grewal KS, Malkowski MJ, Piracha AR, Astbury JC, Kramer CM, Dianzumba S, Reichek N (2000). Effect of general anesthesia on the severity of mitral regurgitation by transesophageal echocardiography. Am J Cardiol.

[10] Alachkar MN, Kirschfink A, Grebe J (2022). General anesthesia leads to underestimation of regurgitation severity in patients with secondary mitral regurgitation undergoing transcatheter mitral valve repair. J Cardiothorac Vasc Anesth.

[11] (2024). Transesophageal echocardiography in the evaluation of mitral valve disease. https://www.uptodate.com/contents/transesophageal-echocardiography-in-the-evaluation-of-mitral-valve-disease.

[12] Robinson S, Ring L, Augustine DX (2021). The assessment of mitral valve disease: A guideline from the British Society of Echocardiography. Echo Res Pract.

[13] Meucci MC, Delgado V (2022). Preoperative assessment of mitral valve regurgitation with two- and three-dimensional transesophageal echocardiography. Cir Cardiovasc.

[14] Sanfilippo F, Johnson C, Bellavia D (2017). Mitral regurgitation grading in the operating room: A systematic review and meta-analysis comparing preoperative and intraoperative assessments during cardiac surgery. J Cardiothorac Vasc Anesth.

[15] Khatib D, Methangkool EK, Rong LQ (2023). Preprocedural transesophageal echocardiography recommendations for mitral structural heart interventions: Implications for the cardiac anesthesiologist. J Cardiothorac Vasc Anesth.

[16] Patzelt J, Ulrich M, Magunia H (2017). Comparison of deep sedation with general anesthesia in patients undergoing percutaneous mitral valve repair. J Am Heart Assoc.

[17] Byrne JG, Aklog L, Adams DH (2000). Assessment and management of functional or ischaemic mitral regurgitation. Lancet.

[18] Cohn LH, Rizzo RJ, Adams DH, Couper GS, Sullivan TE, Collins Jr JJ, Aranki SF (1995). The effect of pathophysiology on the surgical treatment of ischemic mitral regurgitation: Operative and late risks of repair versus replacement. Eur J Cardiothorac Surg.

[19] Dion R, Benetis R, Elias B (1995). Mitral valve procedures in ischemic regurgitation. J Heart Valve Dis.

[20] Han Y, Peters DC, Salton CJ (2008). Cardiovascular magnetic resonance characterization of mitral valve prolapse. JACC Cardiovasc Imaging.

